# A comparison of clinical features between neurobrucellosis and tuberculous meningitis

**DOI:** 10.1186/s12883-024-03631-1

**Published:** 2024-04-25

**Authors:** Yueli Zou, Liqing Wang, Yi Li, Yaning Wu, Junying He, Xiujun Yu

**Affiliations:** Department of Neurology, The Second Hospital of Hebei Medical University, Shijiazhuang, 050000 Hebei PR China

**Keywords:** Neurobrucellosis, Tuberculous meningitis, Clinical features

## Abstract

**Backgroud:**

This study aims to compare the clinical manifestations, imaging findings, routine tests, biochemistry indicators and cerebrospinal fluid cytology between neurobrucellosis and tuberculous meningitis. The objective is to evaluate the similarities and differences of these two diseases and improve early diagnosis.

**Methods:**

A comprehensive evaluation was conducted by comparing clinical data, imaging results, routine tests findings, biochemistry indicators and cerebrospinal fluid cytology of patients admitted to the Department of Neurology, the Second Hospital of Hebei Medical University from 2019 to 2021. Statistical analysis was applied to identify significant differences and similarities between the two diseases.

**Results:**

Preliminary analysis demonstrated both diseases commonly present with symptoms such as fever, headache. However, there were no statistical differences between neurobrucellosis and tuberculous meningitis in early clinical data, imaging results, routine tests findings, biochemistry indicators. Further analysis indicates there is a statistically significantly difference in the lymphocyte ratio and neutrophil ratio in the cerebrospinal fluid between the two groups.

**Conclusions:**

Neurobrucellosis and tuberculous meningitis share similarities in early clinical manifestations, imaging findings and initial cerebrospinal fluid parametes, making early-stage differentiation challenging. The ratio of lymphocytes and neutrophil in the cerebrospinal fluid and a detailed medical history investigation can provide clues for early clinical diagnosis. So the examination of CSF cytology might be a potential to distinguish these two diseases and become a powerful tool in the future.

## Introduction

Neurobrucellosis and tuberculous meningitis are both serious neurological infections that pose diagnostic challenges. Due to the absence of distinctive clinical features, misdiagnosis with other infections is common. The diagnostic criteria for neurobrucellosis include cerebrospinal fluid (CSF) agglutination test, enzyme-linked immunosorbent assay (ELLSA), 16s rRNA sequencing and the presence of positive CSF oligoclonal bands [1–3]. However, establishing a diagnosis can be challenging due to potential false-negative results in immunological testing. Nucleic acid amplification tests, acid-fast bacilli smear and culture are widely considered as the gold standard for diagnosing tuberculous meningitis [4, 5]. However, the quantity of CSFs, the timeliness of sending samples for analysis, and the experience of the inspectors resulted in a low positive rate of acid-fast staining. Culure also has the disadvantages of long cycles and low sensitivity [6, 7]. Either of the disease can present with subacute onset, and their early clinical manifestations may be quite similar, making differentiation difficult. Furthermore, both diseases have variable manifestations including arthritis and spondylitis for neurobrucellosis and including tuberculoma, arachnoiditis, myelitis for tuberculous meningitis [7, 8]. Currently, there is lack of literature specifically comparing the clinical features of these two diseases. Understanding the clinical features of these diseases is crucial for accurate and timely diagnosis. By comparing the clinical manifestations, imaging findings, routine tests, biochemistry indicators and cerebrospinal fluid cytology, we can highlight the distinctive characteristics of each disease, contributing to better diagnosis.

## Methods

Patients clinically diagnosed as neurobrucellosis and tuberculous meningitis in the Department of Neurology, the Second Hospital of Hebei Medical University from January 2019 to December 2021 were collected. Incorporated cases were all confirmed by metagenomic next-generation sequencingis of CSF in this study. The CSF samples were all tested for PACEseq metagenomic next-generation sequencingis detection (Hugobiotech, Beijing, China). A positive metagenomic next-generation sequencingis result was considered when at least 1 unique read was mapped to species level and consistant with clinical symptoms. In addition to this confirmation, general data, clinical manifestations and auxiliary examination data were further gathered. This comprehensive data collection included symptoms, imaging results, WBC count in cerebrospinal fluid from the initial lumbar puncture, as well as the biochemical and cytological classification of CSF, enabling a thorough comparison of the distinctions between the two diseases.

In this study, data processing was conducted using SPSS25.0 statistical software. For measurement data conforming to normal distribution were represented as mean ± standard deviation; The T-test was utilized to compare the the groups. For measurement data that did not follow a normal distribution were presented as median and interquartile range. The comparison was performed using Mann-Whitney test and count data was represented by the number of cases. Chi-square test or Fisher exact probability method were used for comparison between the two groups. A significance of *P* < 0.05 was considered statistically significant.

## Results

### General characteristics of the patients

A total of 212 patients were clinically diagnosed with neurobrucellosis and tuberculous meningitis at the Department of Neurology, the Second Hospital of Hebei Medical University from January 2019 to December 2021. Among these, 11 patients with tuberculous meningitis were confirmed by metagenomic next-generation sequencing, while 10 patients with neurobrucellosis were included in this study. Among the tuberculous meningitis cases, there were 9 males and 2 females. The age of onset ranged from 28 to 69 years old, with an average age of 47 years. In the neurobrucellosis group, there were 6 males and 4 females. The onset age ranged from 15 to 70 years old, with an average age of 40 years old. There were no significant differences observed in terms of age (*P* value:0.387) and sex (*P* value:0.361) between the two groups (Table 1).

**Table 1:** Comparison of the two groups

	Tuberculous meningitis	Neurobrucellosis	*P* value
Age	47	40	0.387
Sex			0.361
Male	9	6	
Female	2	4	
Presentation			
Headache	9	6	0.36
Fever	10	9	1.00
Decreased consciousness	4	2	0.63
Cranial nerve involvement	2	3	0.47
meningeal irritatation sign	4	2	0.63
Imaging finding			
Parenchymal involvement	10	7	0.31
Meningeal involvement	2	4	0.36
Hydrocephalus	3	1	0.59
Duration from onset of symptoms to lumbar puncture	23.50 (8.75,49.50)	16.00(10.75,33.00)	0.37
Cerebrospinal fluid Findings			
Leucocyte count	198.80 ± 110.55	266.00 ± 102.32	0.32
Protein level	1.51 ± 0.23	2.14 ± 0.54	0.31
Glucose level	2.07 ± 0.44	2.32 ± 0.16	0.60
Chloride level	116.80(108.80,121.93)	113.00(111.02,116.03)	0.42
Lymphocyte ratio	63%±29%	89%±7%	0.02
Neutrophil ration	37%±33%	10%±6%	0.03

### Clinical features

Among the 11 patients with tuberculous meningitis, 10 experienced fever, 9 had headache, 4 showed sign of meningeal irritation, 4 had disturbance of consciousness and 2 presented with cranial nerve damage. Additionally, 4 of these cases had tuberculous meningitis combined with cerebral infarction. For the 10 patients diagnosed with neurobrucellosis, 9 had fever, 6 experienced headaches, 2 showed positive signs of meningeal irritation, 3 reported dizziness, 2 had limb weakness, 2 had disturbance of consciousness, and 3 presented with cranial nerve damage, one case also exhibited joint pain, tinnitus, peripheral neuropathy. Notably, one case of neurobrucellosis was complicated with cerebral infarction and another case involved a cerebral abscess. There was no significant difference in major clinical manifestations between the two groups (Table 1).

### Imaging examination

All patients in the study underwent magnetic resonance imaging(MRI) scan. Among the 11 patients with tuberculous meningitis, 6 underwent additional MRI enhancement scan. In this group, 10 patients exhibited brain parenchyma involvement, 2 showed meningeal enhancement, and 3 had hydrocephalus. Among the 10 patients with neurobrucellosis, 6 underwent further MRI scan. Within this group, 7 patients had brain parenchyma involvement, 4 showed meningeal enhancement, and 1 had hydrocephalus. There were no statistical significant differences observed between the two groups in terms of MRI findings (Table 1).

### Results of cerebrospinal fluid

After the admission, the first lumbar puncture examinations were performed on all patients, providing insights into the CSF parameters. There were no statistical significant differences observed between the two groups of the duration from onset of symptoms to lumbar puncture (Table 1). In patients with tuberculous meningitis, the average WBC count in the CSF was 198.80 ± 110.55*10^6^/L, the protein content was 1.51 ± 0.23 g/L, and the glucose content was 2.07 ± 0.44mmol/L. The chloride content was recorded as 116.80mmol/L (108.80,121.93mmol/L). The CSF cytology classification showed a median lymphocyte ratio of 63%±29% and a median neutrophil ratio of 37%±33%. In patients with neurobrucella, the mean WBC count in CSF was 266.00 ± 102.32*10^6^/L, the mean protein content was 2.14 ± 0.54 g/L, the.

glucose content was 2.32 ± 0.16mmol/L, and the chloride content was recorded as 113.0mmol/L(111.02,116.03mmol/L). The CSF cytology classification showed a median lymphocyte ratio of 89%±7% and a median neutrophil ratio of 10%±6%. However, there were no statistically significant differences observed between the two groups in terms of CSF number of cells, protein level, glucose level and chloride contents. It is worth mentioning that there were statistically significant differences observed in the ratio of lymphocytes and neutrophil in the CSF. (Table 1).

### Specificity test

In the neurobrucellosis group, a Tiger Red Plate Agglutination Test was conduced on 4 patients, and 3 patients tested positive for the presence of brucellosis. Additionally, a Brucellosis Agglutination Test was performed on 6 patients, and 4 patients tested positive. Furthermore, in one patient’s blood culture, brucella was detected.

## Discussion

Tuberculosis and brucellosis both classified as Class B infectious diseases. Both are great mimickers with multisystemic involvement [9–11], when these infections involve the central nervous system, they can lead to neurobrucellosis and tuberculous meningitis respectively. The most fatal form of tuberculosis, tuberculous meningitis. Occurs in 1–5% of those with tuberculosis [12]. While the incidence of neurobrucellosis in brucellosis is about 4% [13]. In the early stage of these disease, symptoms such as fever, headache and sometimes nausea and vomiting may be present. However, these clinical manifestations lack of specificity, making early diagnosis challenging. Further, previous study showed that Thwaites and Lancet scoring system for diagnosis of tuberculous meningitis falsely identified neurobrucellosis patients as tuberculous meningitis [14, 15]. Culture is the gold standard, but its sensitivity is low. Serology assays are crucial for diagnosis of brucellosis. The most used nowadays are serum agglutination test and ELISA. Serum agglutination test has been used for years and titers more than 1 : 160 are considered positive in nonendemic areas, while titers more than 1 : 320 are positive in endemic zones [16]. Agglutination test in CSF is more significant for diagnosis of neurobrucellosis with highly sensitive and specific by using a cutoff ≥ 1:8 [17]. CSF acid fast bacilli smear is the most widely accessible and affordable rapid diagnostic test for tuberculous meningitis. But the sensitivity of CSF acid fast bacilli smear were only 8% [18]. Recently, metagenomic next-generation sequencing has emerged as a sensitive technology capable of detecting brucella and Mycobacterium tuberculosis from CSF [19, 20]. However, the limited availability and high cost of advanced sequencing techniques hinder its widespread utilization in economically disadvantaged regions.

To improve early recognition of these diseases, this study aims to compare the clinical manifestations, imaging findings, routine laboratory tests, biochemistry parameters and cytology features of neurobrucellosis and tuberculous meningitis diagnosed using metagenomic next-generation sequencing. By identifying similarities and differences between these two conditions, this research aims to facilitate early and accurate diagnosis of these disease.

In terms of early clinical manifestations, the tuberculous meningitis group primarily presented with fever, headache and disturbances in consciousness. In the neurobrucellosis group, the most common clinical manifestations were fever, headache, nausea and vomiting. The presence of VIII cranial nerve involvement favors neurobrucellosis has been reported in many other studies [21, 22]. Consistent with the view, there were 3 presented with cranial nerve (VIVIIVIII) involved in the neurobrucellosis group and 2 (VIVII) in the tuberculous meningitis group. A study by Linda et al. [23] reported the most common complication of neurobrucellosis in adults was hepato/splenomegaly and only one case was found in this group. In a word, there was no statistical difference in the main clinical manifestations between the two groups. Therefore, it is difficult to distinguish between the two diseases based solely on early clinical symptoms. To aid in diagnosis, it is crucial for clinicians to inquire about the medical history of the patients. Gathering information about epidemiological history, tuberculosis contact history, or history of patients with tuberculous meningitis can provide valuable insights. Similarly, for brucellosis, inquiring about contact history with cattle and sheep or consumption of unsterilized milk or milk products can be helpful in establishing the diagnosis.

Imaging studies play an important role in the diagnosis of central nervous system diseases. Tuberculous meningitis is characterized by enhanced meninges at the base of the skull, cerebral infarction, hydrocephalus and tuberculoma, either appearing alone or in combination [24]. The main imaging manifestations of neurobrucellosis were nonspecific [25]: inflammation, vascular damage, basal meningeal enhancements, cranial nerve involvements and white matter damage. In the study, There was no statistical difference between the imaging manifestations of the two groups, indicating that distinguishing between the two diseases based solely on imaging findings can still be challenging.

The examination of CSF is indeed crucial for the differential diagnosis of infectious diseases affecting the central nervous system. CSF cytology test offers outstanding advantages in distinguishing between bacterial, viral and fungal infections [26]. Patients with bacterial meningitis had a predominant neutrophils response in CSF cytology, viral encephalitis had a predominant lymphocytic response in CSF cytology and mixed cell response in cryptococcal meningitis. Further, cryptococcus can be found directly by CSF cytology test with cryptococcal meningitis, other central nervous system infectious diseases cannot directly observe the pathogen in CSF by CSF cytology test. It has been reported that the cytology of CSF both in tuberculous meningitis and in neurobrucellosis are primarily characterized by lymphocytosis accompained by a variable number of neutrophils [21, 27]. Although, in this particular study, no statistical difference was found between tuberculous meningitis and neurobrucellosis in terms of CSF leucocyte count, protein level, glucose and chloride content. There were statistically significant differences observed in the ratio of lymphocytes and neutrophil by cerebrospinal fluid cytology. In order to exclude the effect of the disease course on the changes of the CSF cytology test, we conducted a statistical analysis to compare the time of lumbar puncture between the two groups. No statistically significant differences were observed between in the duration from symptom onset to lumbar puncture. Therefore the data between the two groups are comparable. In this group, the cerebrospinal fluid cytology of neurobrucellosis showed predominantly increased lymphocytes with a certain proportion of neutrophils, similar to the features observed in the tuberculous meningitis group, which is in line with the report of literatures [21, 22, 27]. However, there is significant difference in the ratio of lymphocytes and neutrophils between the groups of tuberculous meningitis and neurobrucellosis. A higher proportion of neutrophils may indicate a greater likelihood of tuberculous meningitis. The possible mechanism is neutrophils readily infiltrated infection foci in tuberculosis, and it plays an important role in swallowing and destroying microorganisms [28]. However, it is reported [29] Brucella-infected neutrophils functions as “Trojan horse” vehicles for bacterial dispersal and as modulators of the Th1 adaptive immunity in infection but barely induce neutrophils activation. In a word, the cerebrospinal fluid cytology may be a low cost and broad availability method compared to the advanced laboratory techniques in distinguishing the diseases. In the future research, it is necessary to expand the number of patients to distinguish the cut-off value of neutrophil ratio between the two diseases.

In conclusion, this study has contributed to a better understanding of the early stage of neurobrucellosis by comparing its clinical data with that of tuberculous meningitis. The overlapping symptoms between these two diseases pose challenges in making a definitive diagnosis, particularly for doctors in non-epidemic areas who may inadvertently misdiagnose or overlook cases resembling tuberculous meningitis. Therefore, timely identification of neruobrucellosis and tuberculous mengingitis is curcial. The ratio of lymphocytes and neutrophils by cerebrospinal fluid cytology can provide valuable clue for clinical diagnosis. A higher proportion of neutrophils may indicate an increased likelihood of tuberculous meningitis; however, further analysis of additional data is required to determine the accurate cut-off value for neutrophils ratio between neruobrucellosis and tuberculous mengingitis. Therefore, the examination of CSF cytology holds potential in distinguishing between these two diseases and could become a powerful diagnostic tool in the future.

## Data Availability

The datasets generated and/or analysed during the current study are not publicly available due to limitations of ethical approval involving the patient data and anonymity but are available from the corresponding author on reasonable request.

## Abbreviations

MRI: Magnetic resonance imaging
CSF: Cerebrospinal fluid
WBC: White Blood Cells
